# A Strong Supporter: Evidence for the Role of the Fifth Finger in Habitual Gripping Activity

**DOI:** 10.1002/ajpa.70205

**Published:** 2026-02-04

**Authors:** Cora Leder, Sarah A. Schrader

**Affiliations:** ^1^ Laboratory for Human Osteoarchaeology, Faculty of Archaeology Leiden University Leiden the Netherlands

**Keywords:** “validated entheses‐based reconstruction of activity” method, entheseal attachment sites, fifth finger, habitual activity

## Abstract

**Objectives:**

The fifth finger plays a key role in manual dexterity, yet its habitual use and functional integration within the hand remain poorly understood. This study investigates the contribution of the fifth ray to habitual gripping activities and its synergistic relationship with the thumb.

**Materials and Methods:**

The “Validated Entheses‐based Reconstruction of Activity” (VERA) method and multivariate statistical analyses were applied to the hand bones of 43 adult male/probable male individuals from three post‐Medieval skeletal collections in the Netherlands. Principal component and pairwise correlation analyses were used to assess covariation among entheseal attachment sites, with particular focus on the opponens digiti minimi (ODM) and third palmar interosseous (PI3).

**Results:**

Both analyses reproduced functional patterns established in previous VERA studies that distinguish precision from power gripping. The ODM clustered with thumb muscles involved in precision grasping, especially the opponens pollicis (OP), suggesting habitual coordination between the fifth finger and thumb. In contrast, the PI3 formed an independent axis of variation and showed weak correlations with other entheses once overall size effects were removed.

**Discussion:**

The findings indicate that the fifth finger functions as a stabilizing and supportive element across both grip types, contributing to object control and manual stability through its opposition to the thumb and flexion at the MCP joint. This study underscores the functional significance of the fifth ray in habitual manual activity and highlights the value of size‐adjusted VERA analyses for detecting subtle patterns of hand use in past populations.

## 1 Introduction

The human hand has long been the subject of bioarchaeological investigations, as it is one of the defining features of our species (Kivell et al. 2016). Research into habitual manual activity, specifically, is becoming increasingly popular as a means of gaining insight into past human behaviors. Most of this research, however, focuses on the thumb and index finger, leaving the rest of the hand understudied (Lemelin and Schmitt 2016). This is despite existing evidence suggesting that the fifth ray of the hand, including the metacarpal and the phalanges, plays an important role in gripping activity.

The fifth ray is the second most mobile ray of the hand, after the thumb, with a range of motion of about 20 to 25 degrees at the carpometacarpal joint (Moran and Berger 2003; Tubiana 1988). This increases the span at which objects can be grasped and allows for proper placement of the fifth finger, often in direct opposition to the thumb, where it can exert the necessary external force for a secure grip (Domalain et al. 2017; Moran and Berger 2003). Biomechanical research has shown that restriction of the fifth finger's phalanges leads to a reduction in grip strength by around 33% (Methot et al. 2010). Similarly, traumatic hand injuries that result in loss of the fifth finger consistently lead to significant loss in overall grip strength in affected patients, while losing a second, third, or fourth finger can be compensated for much more easily (Høgh and Hooper 1988; Robins 1961). Patients who receive reconstructive surgery where another digit is transposed to the fifth finger's place have been shown to regain more grip strength and a wider range of gripping abilities than those who do not receive it (Moran and Berger 2003).

Experimental archaeology research shows that the fifth ray is also heavily recruited during stone tool production (Key et al. 2019). The opposition of the fifth ray and the thumb is particularly important during the propulsion phase of knapping and for the proper execution of precise strikes (Faisal et al. 2010; Fedato et al. 2020; Key et al. 2019; Marzke and Shackley 1986). At the same time, the fifth finger itself is usually placed underneath the point of impact on the core (Fedato et al. 2020; Key et al. 2019; Patiño et al. 2017). In this position, it absorbs the shock of the strike and maintains the core's stability (Key et al. 2019). The fifth finger is also crucial for the manipulation and adjustment of the core between strikes, ensuring proper placement and maximum grip strength (Key et al. 2019; Marzke et al. 1998). Signs of frequent and continuous strain on the fifth ray have been found through increased concentrations of trabecular bone volume underneath the attachment site of the opponens digiti minimi (ODM) muscle in post‐Neolithic agricultural and industrial individuals and among foragers and hunter‐gatherers (Stephens et al. 2018). This suggests that the fifth ray's role in manual activities deserves further investigation.

Studies into muscle synergies during activities such as gripping have recently become possible due to the new “Validated Entheses‐based Reconstruction of Activity” (VERA) method. So far, it has primarily been used to study the use of the hand, specifically (Karakostis et al. 2017; Karakostis and Harvati 2021; Karakostis and Lorenzo 2016). VERA is based on the analysis of entheseal attachment sites, the areas where muscles or ligaments attach to the bone. Changes to these areas have long been considered indicators of stress through repeated activity (Jurmain et al. 2011; Villotte 2009; Villotte and Knüsel 2013), though their use as such has been the subject of some debate. Previous research has largely focused on entheseal surface changes or on size comparisons between single entheses. The former approach has been criticized due to a lack of clear information on the etiology of these entheseal changes, which appears to be multifactorial and influenced by factors such as body size, age, disease, and anatomical variation (Foster et al. 2012; Henderson and Alves Cardoso 2013). Several studies have also failed to show a direct correlation between enthesis size and muscle size or activity (see e.g., Wallace et al. 2017; Williams‐Hatala et al. 2016). In order to control for these issues, VERA makes use of the multivariate proportional relationships between entheses within each individual, instead of comparing the sizes of single entheses between individuals (Karakostis et al. 2017; Karakostis and Harvati 2021; Karakostis and Lorenzo 2016). In short, VERA relies on 3D surface imaging to isolate and measure entheseal attachment sites. Multivariate statistical analysis like PCA is then used to identify activity patterns across muscle groups, such as clear differentiation between muscles involved in power gripping versus precision gripping (Karakostis 2023; Karakostis and Harvati 2021). The method has been experimentally validated through animal studies (Karakostis, Jeffery, and Harvati 2019; Karakostis, Wallace, et al. 2019; Karakostis and Wallace 2023) and has been successfully used to distinguish between different types of habitual gripping activity in archaeological populations (Karakostis et al. 2017, 2018; Karakostis and Lorenzo 2016). While most applications of VERA have focused on the thumb and first finger, one study found pronounced differences in the use of the fifth finger between highly specialized and less specialized female early industrial workers (Karakostis and Hotz 2022). However, presently, no research into the precise role(s) of the fifth ray in habitual manual activity exists.

Here, we present the results of a study, which, for the first time, focuses explicitly on better understanding the role of the fifth finger in power and precision gripping activity. Using the VERA method and multivariate analyses, we aim to explore the use of the fifth ray of the hand during habitual grasping activities and especially its relationship with the thumb. We purposefully study the fifth ray in combination with the thumb, due to their relationship as antagonists, which is crucial to the proper execution of many gripping activities (Høgh and Hooper 1988; Key et al. 2019; Lemelin and Schmitt 2016; Tubiana et al. 1998). The focus of this study lies on the entheses of two muscles of the fifth ray: the ODM and the third palmar interosseous (PI3). With previous VERA studies having differentiated between precision and power gripping, we hypothesize that:

1. We will find a similar pattern based on the previously studied entheses within our individuals.
2. The ODM will likely show an association with the precision gripping pattern based on the existing analysis of ODM among female early industrial workers by Karakostis and Hotz (2022).
3. The PI3 could be associated with the power grasping group, due to its biomechanical role in flexing the MCP joint and pulling the fifth finger towards the middle of the hand (Hirt et al. 2016; Liss 2012).

## 2 Materials and Methods

For this study, a selection of individuals from three post‐Medieval sites in the current‐day Netherlands was chosen. All three skeletal collections originate from graveyard contexts, with burials dating between the early 1600s and the mid‐1800s. They include the rural dairy farming community of Middenbeemster, factory workers from the urban lower‐class graveyard of Arnhem, and the skeletal collection from the higher middle‐class urban site of Zwolle (De Vries 1961; Ten Hove 2005; Aten 1992). Access to these individuals was granted in accordance with the ethical guidelines of the Laboratory for Human Osteoarchaeology at Leiden University, where the collections are housed.

Sex and age‐at‐death were estimated using standard osteoarchaeological methods. Biological sex was estimated using morphological features of the pelvis and the skull (Buikstra and Ubelaker 1994). Age‐at‐death was estimated based on ectocranial suture closure (Meindl and Lovejoy 1985), and age‐related changes to the pubic symphysis (Brooks and Suchey 1990) and to the auricular surface (Buckberry and Chamberlain 2002). From the three groups, we selected 43 individuals that had been assigned “male” or “probable male”, with estimated ages‐at‐death between 19 years and 50+ years (Table 1). We chose male/probable male individuals to narrow the range of habitual activities and eliminate potentially confounding sex‐based differences in entheseal changes (Milella et al. 2012; Santana‐Cabrera et al. 2015). While EC studies generally exclude individuals over the age of 50 entheses (Milella et al. 2012), previous VERA studies suggest that age‐at‐death does not significantly impact the results of the multivariate analyses and does not affect the observed grasping patterns (Karakostis 2023; Karakostis et al. 2017; Karakostis and Lorenzo 2016). Only individuals without signs of progressive degenerative diseases or other potentially confounding pathologies such as osteoarthritis, rheumatoid arthritis, or gout, and with preserved first and fifth metacarpals and proximal phalanges of the right hand were included.

**TABLE 1:** Demographic composition of the individuals included in this study.

	Young adult	Middle adult	Old adult	Total
Arnhem	6	10	3	19
Middenbeemster	7	7	3	17
Zwolle	2	4	1	7

A total of eight muscle insertion sites were examined. Based on previous VERA studies, the entheses of the opponens pollicis (OP), abductor pollicis/flexor pollicis brevis (ABP/FPB), adductor pollicis (ADP), extensor pollicis brevis (EPB), and abductor/flexor digiti minimi (ADM/FDM) were included. The ABP and FPB, as well as the ADM and FDM share insertion sites, which is why they have been grouped together in accordance with other VERA studies (see e.g., Karakostis et al. 2017; Karakostis and Lorenzo 2016). The OP, ABP/FPB, and ADP have previously been associated with precision gripping activity, where an object is held between the pads of the thumb and other fingers, or between the pad of the thumb and the lateral aspect of another finger, usually the second (Long et al. 1970; Maier and Hepp‐Reymond 1995). The EPB and ADM/FDM have been associated with power gripping, where the hand clamps around an object, with the thumb providing counterpressure to the other fingers and the palm (Karakostis et al. 2018; Karakostis and Lorenzo 2016; Kunze et al. 2022; Napier 1956; Park et al. 2014). In addition, we have included the abductor pollicis longus (APL). Its role is to abduct and extend the thumb at the base of the metacarpal bone, such as when spreading the fingers around a large object (Hirt et al. 2016). Being heavily involved in the movement and stabilization of the thumb (Hirt et al. 2016; van Oudenaarde 1991), it plays a role in the positioning of the thumb in opposition to the fifth finger when using power grasps on larger objects, making it a relevant addition to the selected entheses. Two entheses of the fifth ray were chosen as the main subjects of this study: the insertion sites of the ODM and of the third palmar interosseous muscle (PI3). The ODM is responsible for the abduction and opposition of the fifth ray and the flexion of the MCP joint (Hirt et al. 2016; Schreuders et al. 2007) and has recently been shown to play a role in highly specialized habitual tasks in a population with well‐documented life histories (Karakostis and Hotz 2022). The third palmar interosseous muscle is responsible for adducting the fifth proximal phalange and pulling the fifth finger towards the middle of the hand and towards the other fingers (Hirt et al. 2016). These two muscles thus perform opposing actions and could be associated with different manual activity patterns. Table 2 provides an overview of all included entheses.

**TABLE 2:** List of entheseal attachment sites included in this study and the corresponding muscle functions.

Name	Abbreviation	Main function	Location of the enthesis (insertion)
Opponens pollicis	OP	Abduction, rotation, flexion of the thumb at the CMC joint	Distal radial diaphysis of the first MC
Abductor pollicis	ABP	Abduction of the thumb	Radial base of the first proximal phalanx
Flexor pollicis brevis	FPB	Flexion of the first MCP joint	Radial base of the first proximal phalanx
Adductor pollicis	ADP	Adduction and opposition of the thumb	Ulnar base of the first proximal phalanx
Extensor pollicis brevis	EPB	Extension of the thumb at the MCP joint	Dorsal base of the first proximal phalanx
Abductor pollicis longus	APL	Abduction and medial rotation of the thumb at the CMC joint	Radial base of the first MC
Abductor digiti minimi	ADM	Abduction of the fifth digit	Ulnar base of the fifth proximal phalanx
Flexor digiti minimi	FDM	Flexion of the fifth digit	Ulnar base of the fifth proximal phalanx
Opponens digiti minimi	ODM	Rotation and flexion of the fifth ray at the CMC joint	Ulnar diaphysis of the fifth MC
Third palmar interosseous	PI3	Adduction and flexion of the MCP joint	Radial base of the fifth proximal phalanx

### 2.1 Scanning and Data Processing

**FIGURE 1:**
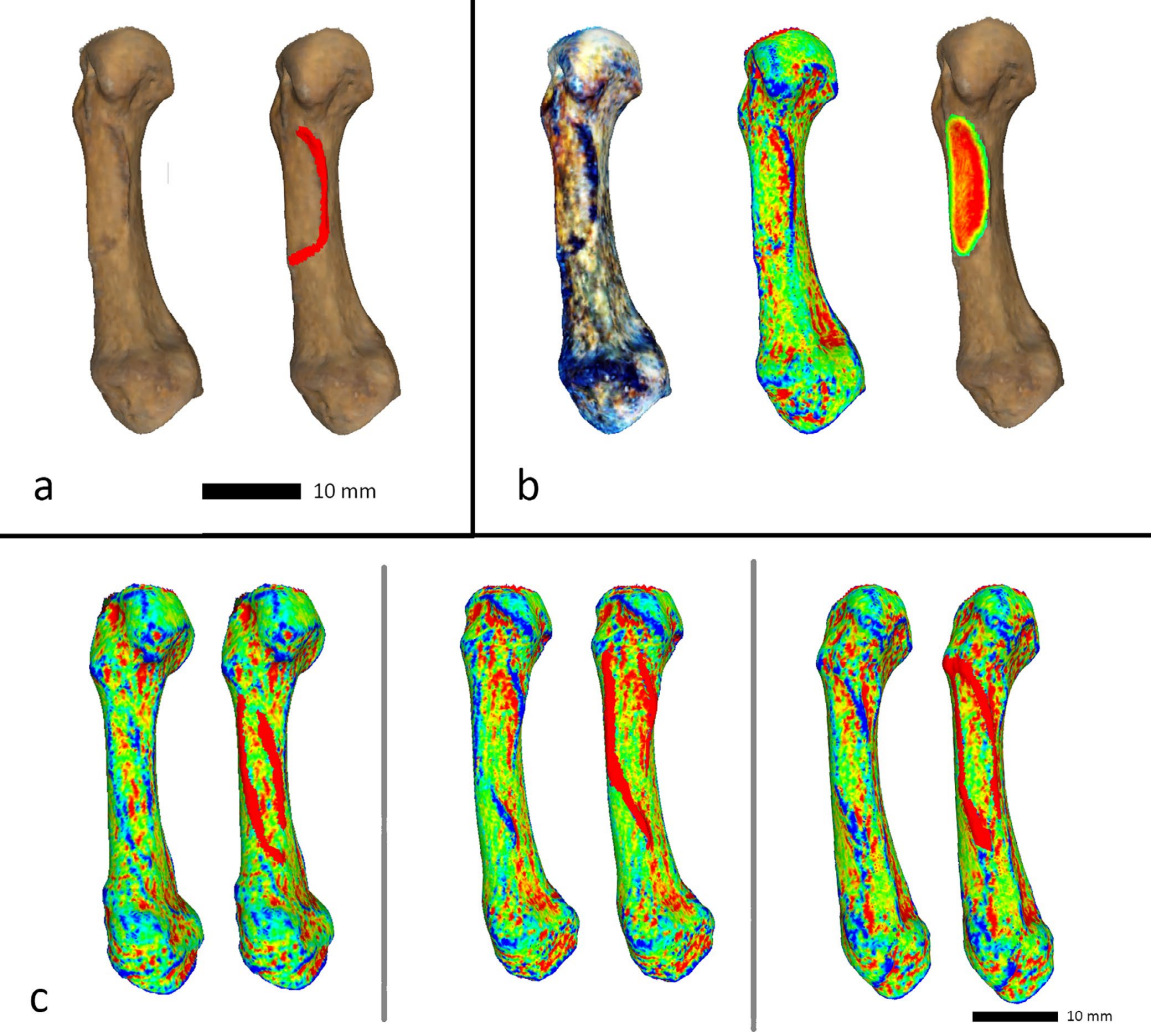
Examples of ODM insertion sites of four different individuals. (a) shows a distinctive ridge along the insertion site on the left side, with the ridge highlighted in red for comparison on the right side. (b) shows the different processing stages of VERA using the same individual (all visual processing performed in Meshlab in accordance with the VERA 1.0 protocol (Karakostis and Harvati 2021; Karakostis 2023)). (c) shows three additional examples of ODM insertion sites after the scans had been processed using the “discrete curvatures” filter in Meshlab. The insertion sites are delineated by elevated ridges that are visible in blue on the left side of each pair (highlighted in red on the right side for easier identification).

3D surface scans of the hand bones were made using a handheld Artec Space Spider scanner (Artec Inc., Luxembourg). The 3D models were saved as Polygon File Format (.ply) files and imported into the open‐source software Meshlab version 2022.02 (ISTI‐CNR, Pisa) for analysis. Analysis took place according to the established VERA protocol, which has previously been described in detail (Karakostis 2023; Karakostis et al. 2018; Karakostis and Harvati 2021; Karakostis and Lorenzo 2016). To summarize, the borders of entheseal attachment sites are determined through a combination of color mapping filters that represent criteria such as changes in surface elevation, color changes, and other irregularities, as well as geodesic distances. The highlighted surface changes are then used to delineate the enthesis and crop it out of the larger image of the bone. The exact surface area of the isolated attachment site can then be calculated in square millimeters (see Figure 1 for examples).

### 2.2 Statistical Analyses

In order to assess the repeatability, four entheses (OP, ADM/FDM, ODM and PI3) were delineated and measured again in 15 randomly chosen individuals after a period of 5 months. These entheses were selected because the latter three represent the fifth ray, and because while OP, ADM/FDM and ODM have previously been evaluated for their inter‐ and intraobserver reliability (see e.g., Karakostis and Lorenzo 2016), the PI3 had not. Paired *t*‐tests were used to test for significant differences (*p* < 0.05) between the two sets of measurements.

Following the existing VERA statistical protocol (Karakostis 2023; Karakostis et al. 2018; Karakostis and Harvati 2021), the data were assessed for multivariate patterns through principal component analysis (PCA). PCA was performed on a correlation matrix due to the variables having different scales. Two PCAs were performed, the first using the raw entheseal sizes and the second using size‐adjusted data. Size adjustment was performed following the “geometric mean” approach, where each raw measurement is divided by the geometric mean of all entheseal measurements for each individual. The outcome was then log‐transformed on the basis of natural logarithms (Almécija et al. 2010; Karakostis et al. 2017; Karakostis and Hotz 2022). No activity groups were assumed prior to the analyses. Scree plots were used to determine the relevant principal components. Before the analyses, we evaluated whether the data met the required statistical assumptions, including normal distribution through histograms and Shapiro–Wilk's tests, and the absence of outliers using *z*‐scores (Harrell Jr and Slaughter 2001; Schreiber 2021). The data's fitness for PCA was assessed using the Kaiser‐Meyer‐Olkin test and Bartlett's test of sphericity on the correlation matrix (Schreiber 2021).

In addition, we quantified linear associations among all entheses measurements using pairwise Pearson correlations on both the raw and size‐adjusted data. We computed the full correlation matrix to quantify linear associations between every pair of variables. For both the raw and size‐adjusted datasets, we calculated correlation coefficients (*r*) with two‐sided *p*‐values and visualized the full matrices as heatmaps using a common color scale (−1 to +1) to facilitate direct comparison. No zeros or missing values were present, so zero‐replacement or imputation was not required. The size‐adjustment was used to remove the global size effect and emphasize relative structure, enabling clearer interpretation of relationships between entheses. All statistical analyses were performed using R (v4.0.3; R Core Team, 2020), RStudio (Posit Team, 2022).

## 3 Results

Table 3 summarizes the descriptive statistics for the entheseal measurements taken in this study. The repeatability analysis did not show a significant difference in measured entheseal sizes between the first and the second rounds of delineation and measurements. All results of the repeatability analysis are reported in Table 4.

**TABLE 3:** Descriptive statistics for all entheseal measurements (in square millimeters).

	OP	ABP/FPB	ADP	EPB	APL	ADM/FDM	ODM	PI3
*N*	43	43	43	43	43	43	43	43
Range	98.8	48.96	53.11	35.06	24.31	34.35	103.88	27.04
Mean	112.37	70.52	55.45	44.59	30.4	59.16	119.71	34.36
Standard error	4.37	2.03	2.09	1.17	0.98	1.39	4.18	1.02
Standard deviation	28.66	13.28	13.7	7.69	6.45	9.12	27.38	6.68

**TABLE 4:** Results of the repeatability analysis showing non‐significant *p*‐values (in bold).

	OP	ADM/FDM	ODM	PI3
*N*	15.0	15.0	15.0	15.0
df	14.0	14.0	14.0	14.0
Mean different	0.1837	−0.0678	0.3429	−0.1154
SD	1.7174	1.9836	2.0539	0.8386
SE	0.4434	0.5122	0.5303	0.2165
*t*	0.4143	−0.1324	0.6465	−0.533
*p*	**0.6849**	**0.8965**	**0.5284**	**0.6024**
95% CI lower	−0.7674	−1.1663	−0.7946	−0.5798
95% CI upper	1.1348	1.0307	1.4803	0.349

### 3.1 Results of the PCA

The statistical analyses showed that the data were normally distributed, devoid of outliers and fit for PCA. Table 5 shows the factor loadings for the two PCAs performed. While the loading values were relatively low overall, they were deemed acceptable for interpretation given that they are in similar ranges (see, e.g., Yong and Pearce 2013 for discussion). In both cases, the first two PCs were plotted.

**TABLE 5:** Factor loadings for all eight variables from both principal component analyses (PCAs) performed.

Principal component		Eigen‐value	% of variance	Factor loadings							
				OP	ABP/FPB	ADP	EPB	APL	ADM/FDM	ODM	PI3
PCA1	PC1	4.099	50.04	0.42	0.44	0.38	0.30	0.21	0.30	0.45	0.23
	PC2	**2.043**	**24.95**	**−0.27**	**−0.22**	**−0.26**	**0.54**	**0.49**	**0.49**	**−0.17**	**−0.11**
PCA2	PC1	**4.372**	**53.4**	**−0.40**	**−0.36**	**−0.32**	**0.44**	**0.36**	**0.41**	**−0.34**	**0.046**
	PC2	1.25	15.3	−0.12	−0.01	−0.28	−0.16	−0.24	−0.14	−0.08	0.895

*Note:* Bold indicates *p* < 0.05.

**FIGURE 2:**
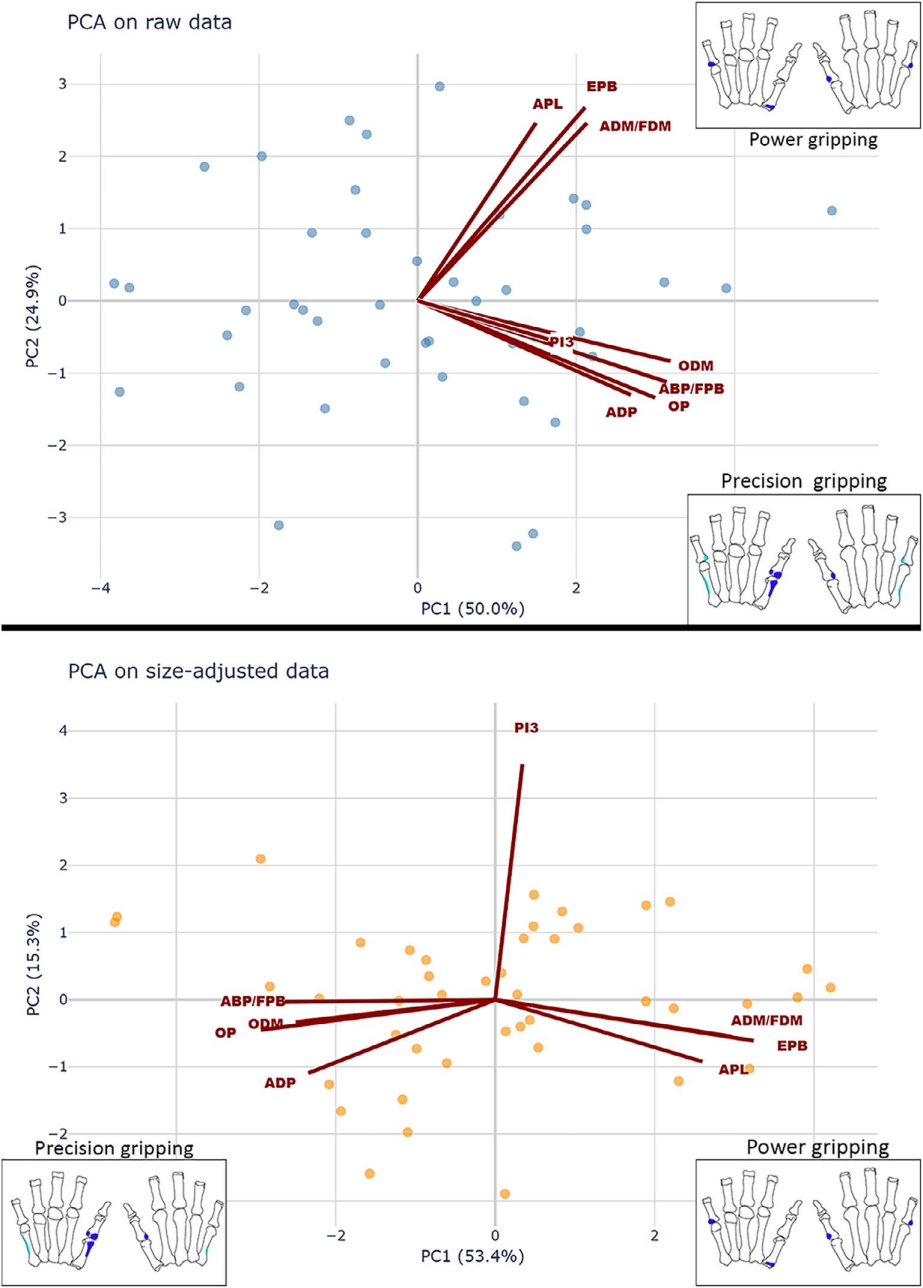
Biplots of the principal component analyses (PCA) on raw entheseal 3D measurements and size‐adjusted measurements across all individuals, including vector loadings for all entheses. Power and precision gripping entheses are marked in blue on the respective illustrations (with the ODM and PI3 in light blue).

For the first PCA (Figure 2), the two first principal components accounted for 74.99% of the variance in the sample. PC1 (accounting for 50.04% of variance) reflects entheseal size with all factor loadings being positive. The ODM and PI3 show similar scores to the other entheses. The second PC accounts for 24.95% of the sample's variance. The three entheses of the OP, ABP/FPB, and ADP have negative loadings, while the entheses of the EPB, APL, and ADM/FDM have positive loading scores. The second PC thus captures a relation in the proportions of these two sets of entheseal measurements. Individuals with proportionally larger entheseal measurements of the first group are represented by negative loadings on PC2, while those with proportionally larger entheseal measurements in the latter set are represented by positive loadings on PC2. Both ODM and PI3 show negative loading scores, associating them with the first group, though the factor loading for PI3 is very low (−0.107).

The second PCA (Figure 2) was carried out on the size‐adjusted data to control for the effect of entheseal size on the patterns observed in PC2 of the previous analysis. After size adjustment, the same pattern can now be observed in PC1. Together, PC1 (53.40%) and PC2 (15.30%) account for 68.70% of the variance. The previously observed pattern is present, with the OP, ABP/FPB, and ADP showing negative loadings. In contrast, the EPB, APL, and ADM/FDM have positive loadings. The loading scores for the ABP/FPB and ADP are very similar to those of the first PCA, while the score for the OP increased notably. The score of the APL decreased, as did the EPB and ADM/FDM scores, though only slightly. The ODM still exhibits a negative loading, though the score has increased notably. The PI3 now has a very low positive loading score (0.046) on PC1. However, PC2 is dominated by the PI3, which shows a very strong positive loading (0.895), whereas all other entheses show small, slightly negative loadings on this axis.

### 3.2 Results of the Correlation Analyses

**FIGURE 3:**
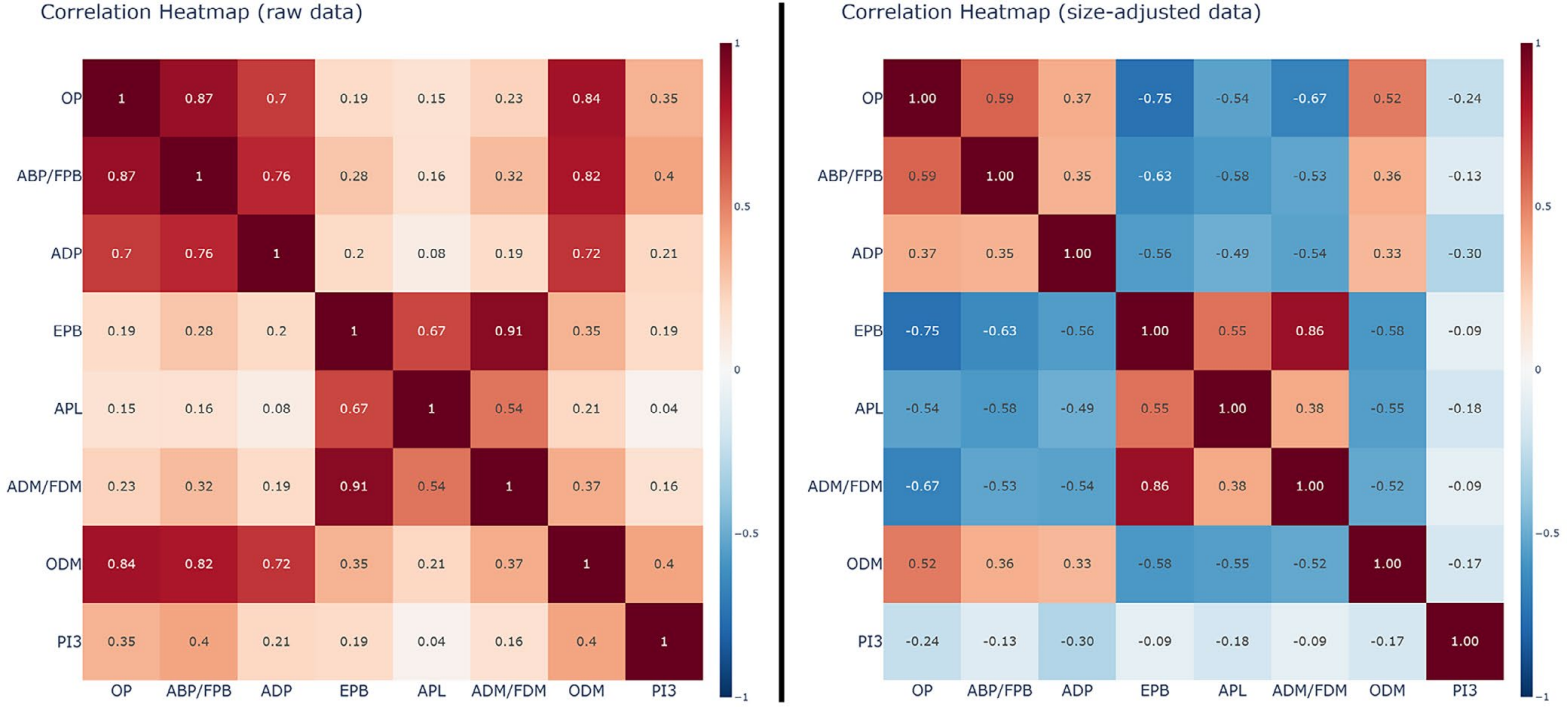
Heatmaps showing the results of the Pearson correlation analysis with correlation coefficients for both raw and size‐adjusted data.

Associations between entheses have been characterized with two complementary Pearson correlation heat‐maps (Figure 3). In the raw (unadjusted) space, all 28 pairwise coefficients are positive (range 0.04–0.91, median *r* = 0.24). The strongest coherence occurs among OP and ABP/FPB (*r* = 0.87), OP and ODM (*r* = 0.84), and ABP/FPB and ODM (*r* = 0.83). The entheses EPB—ADM/FDM also exhibit high covariation (*r* = 0.91), whereas PI3 shows the weakest associations overall (largest |*r*| = 0.40 with ODM).

After size‐adjustment the correlation matrix shifts towards a bipolar structure: 13 of 28 coefficients become negative and the mean absolute correlation increases to 0.52. OP, ABP/FPB, ADP and ODM now form a coherent group (mean within‐group *r* = 0.46) that correlates negatively with the group formed by EPB, APL and ADM/FDM (mean between‐group *r* = −0.61). The largest negative coefficient is OP—EPB (*r* = −0.75), followed by ABP/FPB—EPB (*r* = −0.63) and ODM—EPB (*r* = −0.58). PI3 again displays the weakest associations (largest |*r*| = 0.31 with ADP). Correlation coefficients are reported alongside the heatmaps, and complete numeric correlation tables (r and p for all pairs) are provided in the Supporting Information S1.

## 4 Discussion

Given the apparent importance of the fifth ray for the proper execution of a variety of gripping tasks, it is important to examine it within the framework of entheses‐based reconstruction of habitual manual activity. To this end, the introduction presented three hypotheses, which will be individually discussed below.

Hypothesis 1 *Replication of the precision—power grip pattern*.

The PCA and correlation analyses both provide evidence for two distinct group signatures among the entheses that align with the precision versus power grip framework previously identified in VERA studies. Both raw and size‐adjusted PCAs recover a latent axis that separates the entheses of the muscles involved in precision gripping (OP, ABP/FPB, ADP, including ODM) from those involved in power gripping (EPB, APL, ADM/FDM). In the unadjusted PCA, this axis corresponds to PC2 (28% variance), whereas after size‐adjustment it becomes PC1 (53% variance). These results are congruent with previous VERA studies, replicating the same multivariant pattern among these entheses.

The pairwise correlations reinforce this observation. The analysis of the raw data shows strong correlations between entheses of the same group, especially within the precision grasping group, including the ODM. Associations between the precision and power gripping groups are especially high in the correlation analysis of the raw data. They persist in the size‐adjusted space, though the slight positive correlation between the entheses of the two groups disappears. It is replaced with a contrast pattern that distinguishes two functional groups, confirming the pattern observed in the PCA. Within‐group correlations remain positive and moderate‐to‐strong. Crucially, between‐group coefficients flip sign and become strongly negative. This points towards overall size being an important scaling factor within the raw data that is strong enough to partially obscure the contrasting pattern between the groups. This further reinforces the recommendation to adjust for size in these types of VERA analyses (Karakostis 2023).

Overall, the hypothesized precision—power grip pattern is robustly recovered, especially once size effects are removed.

Hypothesis 2 *ODM as part of the precision gripping system*.

The ODM loads negatively onto the precision—power axis in both raw (loading = −0.18) and size‐adjusted (loading = −0.34) PCA analyses, placing it within the precision gripping cluster. This observation is strengthened by a similar pattern within the correlation analyses, where the ODM strongly correlates with the precision gripping cluster in both the raw and size‐adjusted analyses. Once size is removed, a clear positive correlation with the precision gripping cluster, and especially OP, as well as a negative correlation with the power gripping cluster emerges. The ODM is thus clearly correlated with the entheses that previous VERA studies have classified as being involved in precision gripping, in agreement with our initial hypothesis.

The strongest positive correlation for the ODM being with the OP is in line with the results presented by Karakostis and Hotz (2022), who also observed strong correlations between ODM, ABP/FPB and OP among women engaging in specialized labor. They hypothesize that strongly ODM driven grasping patterns likely reflect “habitual coordination between the fifth ray and the thumb” (Karakosits and Hotz 2022, 12). The strong correlation between the ODM and the entheses of muscles specifically involved in fifth finger—thumb opposition observed in the present study strengthens this assumption. Such opposition occurs during precision gripping and handling of larger objects that involves all fingers, including cradle precision grips where the object rests against the palm (Karakostis and Hotz 2022; Chim 2017; Marzke 1997). Further explorations of the ODM's relationship with other entheses could present an opportunity to better differentiate between occupational groups when studying habitual manual behaviors.

Hypothesis 3 *PI3 as part of the power gripping system*.

PI3 exhibits the weakest correlations with all other muscles and defines its own orthogonal axis in the size‐adjusted PCA (PC2, 15% variance). The correlation analysis of the raw data shows a weak positive correlation with the precision grasping entheses and a weak negative correlation with the power grasping entheses. However, after size‐adjustment, all other entheses show a weak negative correlation with the PI3, suggesting that the initial pattern was mostly a function of overall entheseal sizes. The PI3 is thus not directly associated with either the precision or power gripping groups, or any individual entheses.

Given the synergistic relationships of the muscle systems within the hand, it is unlikely that this simply reflects the PI3 not being involved in the gripping activities performed by the other muscles. On the contrary, existing biomechanical research suggests that the interossei are important participants in both power and precision gripping, as they provide intrinsic balance and stability to the fingers in both their static and dynamic functions (Kozin et al. 1999; Liss 2012; Long et al. 1970). Both the palmar and dorsal interossei further ensure stability of the MCP and IP joints when they experience loading, preventing the collapse of the joints into hyperextension or a “claw” position by acting as antagonists to the extrinsic flexors and extensors of the digits (Liss 2012; Schreuders et al. 2007).

Functionally, in their role as adductors (palmar interossei), abductors (dorsal interossei) and MCP flexors, they are instrumental in the proper positioning of the fingers around an object in power gripping (Eladoumikdachi et al. 2002; Liss 2012; Long et al. 1970). During precision gripping, they are similarly involved during the handling of an object, especially during rotation, which is achieved through adduction and abduction of the digits (Liss 2012; Long et al. 1970). They also provide the necessary compression forces while moving an object towards or away from the palm during precision handling (Long et al. 1970) and contribute substantially to the available grip strength during certain pinch grips such as key pinches (Kozin et al. 1999).

We therefore hypothesize that the divergent PI3 dimension seen in the PCA and the lack of correlations between the PI3 and the other entheses may reflect the overall involvement of the PI3 across both power and precision gripping, not as a main actor but as a supporting structure that stabilizes the fifth finger during either kind of gripping activity. This will have to be explored in future analyses, especially through the inclusion of other interossei. It would be expected that the interossei show similar patterns to the one seen in PI3 above, and that the synergistic relationships among the interossei could be captured through VERA and multivariate analyses such as the ones conducted in this study. Identifying such functional patterns among the entheses of the interossei may aid in improving our ability to better understand past habitual grasping and object handling patterns.

### 4.1 Limitations

The biggest limitation of this study is the lack of available occupational records. While the power and precision of grasping patterns observed in previous VERA studies have been replicated, our interpretations hinge on the accuracy of the underlying grasping patterns, which would ideally be verified with occupational data post‐analysis. In addition, the sample size is limited. Though the sample is around the same size as in comparable VERA studies (e.g., Karakostis et al. 2017; Karakostis and Hotz 2022) it may not be able to represent patterns on a larger scale and has the potential to affect the reliability of the statistical analyses. These are problems that archaeologists must contend with regularly and must be considered carefully when interpreting the results. Finally, the study is limited in that its focus rests on just two of the muscles that govern the use of the fifth ray. The OP and PI3 were chosen because their respective functions suggested that they would be useful for at least an initial study of the fifth ray. This was shown to be an accurate assumption based on the results discussed above, but other entheses should be included in future studies with larger sample sizes.

## 5 Conclusion

Our study presents the results of a direct investigation into the role of the fifth finger in habitual gripping activity in archaeological populations. The study demonstrates that the fifth ray can be meaningfully integrated into VERA‐based reconstructions of habitual manual activity, with clear functional differentiation emerging among its associated entheseal signatures. Both PCA and correlation analyses robustly replicate the precision and power gripping patterns established in previous VERA studies and confirm the hypothesized inclusion of the ODM within the precision gripping system, in particular through its strong association with the OP. By contrast, the PI3 defines an independent axis of variation and shows weak correlations with other entheses, once overall enthesis size is removed from the analyses. These results could reflect a stabilizing role across both power and precision grips, rather than exclusive participation in either. Our findings highlight the importance of size‐adjustment for revealing functional contrasts and suggest that the interossei may constitute a distinct but functionally crucial dimension within grasping systems. While limited by sample size and the absence of occupational data, this study underscores the analytical value of incorporating fifth‐ray musculature into entheses‐based approaches and sets the stage for future research that broadens the range of included muscles and integrates comparative datasets to refine our understanding of past habitual gripping behaviors.

## Author Contributions

**Cora Leder:** conceptualization (lead), data curation (lead), formal analysis (lead), investigation (lead), methodology (lead), software (lead), visualization (lead), writing – original draft (lead). **Sarah A. Schrader:** conceptualization (supporting), methodology (supporting), resources (lead), supervision (lead), validation (lead), writing – review and editing (lead).

## Funding

This work was supported by Aard‐ en Levenswetenschappen, Nederlandse Organisatie voor Wetenschappelijk Onderzoek.

## Supporting information


**Data S1:** Supporting Information.

## Data Availability

The data that support the findings of this study are available from the corresponding author upon reasonable request.
