# Supplementary material for: A Strong Supporter: Evidence for the Role of the Fifth Finger in Habitual Gripping Activity
Source: Am J Biol Anthropol. 2026 Feb 4;189(2):e70205. doi: 10.1002/ajpa.70205 (PMC12873511; doi:10.1002/ajpa.70205)
Supplement: Supplementary file 1 — Data S1: Supporting Information. [file AJPA-189-e70205-s001.docx]

| **Data** | **Variable 1** | **Variable 2** | **r** | **t-value** | **p-value** | **n** | **df** | **Sig.** |
| --- | --- | --- | --- | --- | --- | --- | --- | --- |
| Raw | OP | ABP/FPB | 0.868 | 11.171 | **p<0.001** | 43 | 41 | *** |
| Raw | OP | ADP | 0.701 | 6.299 | **p<0.001** | 43 | 41 | *** |
| Raw | OP | EPB | 0.188 | 1.224 | 0.228 | 43 | 41 |  |
| Raw | OP | APL | 0.146 | 0.947 | 0.349 | 43 | 41 |  |
| Raw | OP | ADM/FDM | 0.235 | 1.545 | 0.13 | 43 | 41 |  |
| Raw | OP | ODM | 0.844 | 10.076 | **p<0.001** | 43 | 41 | *** |
| Raw | OP | PI3 | 0.349 | 2.386 | 0.022 | 43 | 41 | * |
| Raw | ABP/FPB | OP | 0.868 | 11.171 | **p<0.001** | 43 | 41 | *** |
| Raw | ABP/FPB | ADP | 0.755 | 7.38 | **p<0.001** | 43 | 41 | *** |
| Raw | ABP/FPB | EPB | 0.279 | 1.861 | 0.07 | 43 | 41 |  |
| Raw | ABP/FPB | APL | 0.159 | 1.028 | 0.31 | 43 | 41 |  |
| Raw | ABP/FPB | ADM/FDM | 0.322 | 2.181 | 0.035 | 43 | 41 | * |
| Raw | ABP/FPB | ODM | 0.825 | 9.339 | **p<0.001** | 43 | 41 | *** |
| Raw | ABP/FPB | PI3 | 0.399 | 2.785 | 0.008 | 43 | 41 | ** |
| Raw | ADP | OP | 0.701 | 6.299 | **p<0.001** | 43 | 41 | *** |
| Raw | ADP | ABP/FPB | 0.755 | 7.38 | **p<0.001** | 43 | 41 | *** |
| Raw | ADP | EPB | 0.197 | 1.285 | 0.206 | 43 | 41 |  |
| Raw | ADP | APL | 0.084 | 0.537 | 0.594 | 43 | 41 |  |
| Raw | ADP | ADM/FDM | 0.192 | 1.255 | 0.217 | 43 | 41 |  |
| Raw | ADP | ODM | 0.721 | 6.666 | **p<0.001** | 43 | 41 | *** |
| Raw | ADP | PI3 | 0.213 | 1.397 | 0.17 | 43 | 41 |  |
| Raw | EPB | OP | 0.188 | 1.224 | 0.228 | 43 | 41 |  |
| Raw | EPB | ABP/FPB | 0.279 | 1.861 | 0.07 | 43 | 41 |  |
| Raw | EPB | ADP | 0.197 | 1.285 | 0.206 | 43 | 41 |  |
| Raw | EPB | APL | 0.67 | 5.783 | **p<0.001** | 43 | 41 | *** |
| Raw | EPB | ADM/FDM | 0.908 | 13.871 | **p<0.001** | 43 | 41 | *** |
| Raw | EPB | ODM | 0.352 | 2.409 | 0.021 | 43 | 41 | * |
| Raw | EPB | PI3 | 0.191 | 1.247 | 0.22 | 43 | 41 |  |
| Raw | APL | OP | 0.146 | 0.947 | 0.349 | 43 | 41 |  |
| Raw | APL | ABP/FPB | 0.159 | 1.028 | 0.31 | 43 | 41 |  |
| Raw | APL | ADP | 0.084 | 0.537 | 0.594 | 43 | 41 |  |
| Raw | APL | EPB | 0.67 | 5.783 | **p<0.001** | 43 | 41 | *** |
| Raw | APL | ADM/FDM | 0.544 | 4.151 | **p<0.001** | 43 | 41 | *** |
| Raw | APL | ODM | 0.211 | 1.384 | 0.174 | 43 | 41 |  |
| Raw | APL | PI3 | 0.041 | 0.265 | 0.792 | 43 | 41 |  |
| Raw | ADM/FDM | OP | 0.235 | 1.545 | 0.13 | 43 | 41 |  |
| Raw | ADM/FDM | ABP/FPB | 0.322 | 2.181 | 0.035 | 43 | 41 | * |
| Raw | ADM/FDM | ADP | 0.192 | 1.255 | 0.217 | 43 | 41 |  |
| Raw | ADM/FDM | EPB | 0.908 | 13.871 | **p<0.001** | 43 | 41 | *** |
| Raw | ADM/FDM | APL | 0.544 | 4.151 | **p<0.001** | 43 | 41 | *** |
| Raw | ADM/FDM | ODM | 0.373 | 2.573 | 0.014 | 43 | 41 | * |
| Raw | ADM/FDM | PI3 | 0.156 | 1.014 | 0.316 | 43 | 41 |  |
| Raw | ODM | OP | 0.844 | 10.076 | **p<0.001** | 43 | 41 | *** |
| Raw | ODM | ABP/FPB | 0.825 | 9.339 | **p<0.001** | 43 | 41 | *** |
| Raw | ODM | ADP | 0.721 | 6.666 | **p<0.001** | 43 | 41 | *** |
| Raw | ODM | EPB | 0.352 | 2.409 | 0.021 | 43 | 41 | * |
| Raw | ODM | APL | 0.211 | 1.384 | 0.174 | 43 | 41 |  |
| Raw | ODM | ADM/FDM | 0.373 | 2.573 | 0.014 | 43 | 41 | * |
| Raw | ODM | PI3 | 0.404 | 2.825 | 0.007 | 43 | 41 | ** |
| Raw | PI3 | OP | 0.349 | 2.386 | 0.022 | 43 | 41 | * |
| Raw | PI3 | ABP/FPB | 0.399 | 2.785 | 0.008 | 43 | 41 | ** |
| Raw | PI3 | ADP | 0.213 | 1.397 | 0.17 | 43 | 41 |  |
| Raw | PI3 | EPB | 0.191 | 1.247 | 0.22 | 43 | 41 |  |
| Raw | PI3 | APL | 0.041 | 0.265 | 0.792 | 43 | 41 |  |
| Raw | PI3 | ADM/FDM | 0.156 | 1.014 | 0.316 | 43 | 41 |  |
| Raw | PI3 | ODM | 0.404 | 2.825 | 0.007 | 43 | 41 | ** |
| Size-adjusted | OP | ABP/FPB | 0.59 | 4.674 | **p<0.001** | 43 | 41 | *** |
| Size-adjusted | OP | ADP | 0.37 | 2.552 | 0.015 | 43 | 41 | * |
| Size-adjusted | OP | EPB | -0.746 | -7.175 | **p<0.001** | 43 | 41 | *** |
| Size-adjusted | OP | APL | -0.54 | -4.109 | **p<0.001** | 43 | 41 | *** |
| Size-adjusted | OP | ADM/FDM | -0.67 | -5.776 | **p<0.001** | 43 | 41 | *** |
| Size-adjusted | OP | ODM | 0.521 | 3.913 | **p<0.001** | 43 | 41 | *** |
| Size-adjusted | OP | PI3 | -0.235 | -1.548 | 0.129 | 43 | 41 |  |
| Size-adjusted | ABP/FPB | OP | 0.59 | 4.674 | **p<0.001** | 43 | 41 | *** |
| Size-adjusted | ABP/FPB | ADP | 0.35 | 2.396 | 0.021 | 43 | 41 | * |
| Size-adjusted | ABP/FPB | EPB | -0.636 | -5.272 | **p<0.001** | 43 | 41 | *** |
| Size-adjusted | ABP/FPB | APL | -0.577 | -4.521 | **p<0.001** | 43 | 41 | *** |
| Size-adjusted | ABP/FPB | ADM/FDM | -0.528 | -3.979 | **p<0.001** | 43 | 41 | *** |
| Size-adjusted | ABP/FPB | ODM | 0.364 | 2.502 | 0.016 | 43 | 41 | * |
| Size-adjusted | ABP/FPB | PI3 | -0.128 | -0.826 | 0.414 | 43 | 41 |  |
| Size-adjusted | ADP | OP | 0.37 | 2.552 | 0.015 | 43 | 41 | * |
| Size-adjusted | ADP | ABP/FPB | 0.35 | 2.396 | 0.021 | 43 | 41 | * |
| Size-adjusted | ADP | EPB | -0.565 | -4.379 | **p<0.001** | 43 | 41 | *** |
| Size-adjusted | ADP | APL | -0.492 | -3.623 | **p<0.001** | 43 | 41 | *** |
| Size-adjusted | ADP | ADM/FDM | -0.542 | -4.132 | **p<0.001** | 43 | 41 | *** |
| Size-adjusted | ADP | ODM | 0.334 | 2.27 | 0.029 | 43 | 41 | * |
| Size-adjusted | ADP | PI3 | -0.3 | -2.012 | 0.051 | 43 | 41 |  |
| Size-adjusted | EPB | OP | -0.746 | -7.175 | **p<0.001** | 43 | 41 | *** |
| Size-adjusted | EPB | ABP/FPB | -0.636 | -5.272 | **p<0.001** | 43 | 41 | *** |
| Size-adjusted | EPB | ADP | -0.565 | -4.379 | **p<0.001** | 43 | 41 | *** |
| Size-adjusted | EPB | APL | 0.549 | 4.209 | **p<0.001** | 43 | 41 | *** |
| Size-adjusted | EPB | ADM/FDM | 0.865 | 11.015 | **p<0.001** | 43 | 41 | *** |
| Size-adjusted | EPB | ODM | -0.58 | -4.554 | **p<0.001** | 43 | 41 | *** |
| Size-adjusted | EPB | PI3 | -0.093 | -0.596 | 0.554 | 43 | 41 |  |
| Size-adjusted | APL | OP | -0.54 | -4.109 | **p<0.001** | 43 | 41 | *** |
| Size-adjusted | APL | ABP/FPB | -0.577 | -4.521 | **p<0.001** | 43 | 41 | *** |
| Size-adjusted | APL | ADP | -0.492 | -3.623 | **p<0.001** | 43 | 41 | *** |
| Size-adjusted | APL | EPB | 0.549 | 4.209 | **p<0.001** | 43 | 41 | *** |
| Size-adjusted | APL | ADM/FDM | 0.383 | 2.657 | 0.011 | 43 | 41 | * |
| Size-adjusted | APL | ODM | -0.554 | -4.261 | **p<0.001** | 43 | 41 | *** |
| Size-adjusted | APL | PI3 | -0.184 | -1.199 | 0.237 | 43 | 41 |  |
| Size-adjusted | ADM/FDM | OP | -0.67 | -5.776 | **p<0.001** | 43 | 41 | *** |
| Size-adjusted | ADM/FDM | ABP/FPB | -0.528 | -3.979 | **p<0.001** | 43 | 41 | *** |
| Size-adjusted | ADM/FDM | ADP | -0.542 | -4.132 | **p<0.001** | 43 | 41 | *** |
| Size-adjusted | ADM/FDM | EPB | 0.865 | 11.015 | **p<0.001** | 43 | 41 | *** |
| Size-adjusted | ADM/FDM | APL | 0.383 | 2.657 | 0.011 | 43 | 41 | * |
| Size-adjusted | ADM/FDM | ODM | -0.516 | -3.859 | **p<0.001** | 43 | 41 | *** |
| Size-adjusted | ADM/FDM | PI3 | -0.092 | -0.594 | 0.556 | 43 | 41 |  |
| Size-adjusted | ODM | OP | 0.521 | 3.913 | **p<0.001** | 43 | 41 | *** |
| Size-adjusted | ODM | ABP/FPB | 0.364 | 2.502 | 0.016 | 43 | 41 | * |
| Size-adjusted | ODM | ADP | 0.334 | 2.27 | 0.029 | 43 | 41 | * |
| Size-adjusted | ODM | EPB | -0.58 | -4.554 | **p<0.001** | 43 | 41 | *** |
| Size-adjusted | ODM | APL | -0.554 | -4.261 | **p<0.001** | 43 | 41 | *** |
| Size-adjusted | ODM | ADM/FDM | -0.516 | -3.859 | **p<0.001** | 43 | 41 | *** |
| Size-adjusted | ODM | PI3 | -0.174 | -1.134 | 0.263 | 43 | 41 |  |
| Size-adjusted | PI3 | OP | -0.235 | -1.548 | 0.129 | 43 | 41 |  |
| Size-adjusted | PI3 | ABP/FPB | -0.128 | -0.826 | 0.414 | 43 | 41 |  |
| Size-adjusted | PI3 | ADP | -0.3 | -2.012 | 0.051 | 43 | 41 |  |
| Size-adjusted | PI3 | EPB | -0.093 | -0.596 | 0.554 | 43 | 41 |  |
| Size-adjusted | PI3 | APL | -0.184 | -1.199 | 0.237 | 43 | 41 |  |
| Size-adjusted | PI3 | ADM/FDM | -0.092 | -0.594 | 0.556 | 43 | 41 |  |
| Size-adjusted | PI3 | ODM | -0.174 | -1.134 | 0.263 | 43 | 41 |  |
|  |  |  |  |  |  |  |  |  |
| Significance: | * | p<0.05 |  |  |  |  |  |  |
|  | ** | p<0.01 |  |  |  |  |  |  |
|  | *** | p<0.05 |  |  |  |  |  |  |
